# 'Relief of oppression': An organizing principle for researchers' obligations to participants in observational studies in the developing world

**DOI:** 10.1186/1471-2458-10-384

**Published:** 2010-06-30

**Authors:** James V Lavery, Sunita VS Bandewar, Joshua Kimani, Ross EG Upshur, Frances A Plummer, Peter A Singer

**Affiliations:** 1 Centre for Research on Inner City Health & Centre for Global Health Research, Keenan Research Centre, Li Ka Shing Knowledge Institute, St. Michael's Hospital, Toronto, Canada; 2 Department of Public Health Sciences and Joint Centre for Bioethics, University of Toronto, Toronto, Canada; 3 McLaughlin-Rotman Centre for Global Health, University Health Network, Toronto, Canada; 4 Department of Microbiology, University of Nairobi; 5 Joint Centre for Bioethics, Department of Family & Community Medicine, and Department of Public Health Sciences, University of Toronto, Toronto, Canada; 6 Department of Medical Microbiology, University of Manitoba, Winnipeg, Canada; 7 Public Health Agency of Canada, Winnipeg, Canada; 8 Department of Medicine, University of Toronto, Toronto, Canada

## Abstract

**Background:**

A central question in the debate about exploitation in international research is whether investigators and sponsors from high-income countries (HIC) have obligations to address background conditions of injustice in the communities in which they conduct their research, beyond the healthcare and other research-related needs of participants, to aspects of their basic life circumstances.

**Discussion:**

In this paper, we describe **t**he Majengo sexually transmitted disease (STD) Cohort study, a long-term prospective, observational cohort of sex workers in Nairobi, Kenya. Despite important scientific contributions and a wide range of benefits to the women of the cohort, most of the women have remained in the sex trade during their long-standing participation in the cohort, prompting allegations of exploitation. The Majengo STD cohort case extends the debate about justice in international research ethics beyond clinical trials into long-term observational research. We sketch the basic features of a new approach to understanding and operationalizing obligations of observational researchers, which we call 'relief of oppression'. 'Relief of oppression' is an organizing principle, analogous to the principle of harm reduction that is now widely applied in public health practice. Relief of oppression aims to help observational researchers working in conditions of injustice and deprivation to clarify their ethical obligations to participants. It aims to bridge the gap between a narrow, transaction-oriented account of avoiding exploitation and a broad account emphasizing obligations of reparation for historic injustices. We propose that relief of oppression might focus researchers' consideration of benefits on those that have some relevance to background conditions of injustice, and so elevate the priority of these benefits, in relation to others that might be considered and negotiated with participants, according to the degree to which the participating communities are constrained in their realization of fundamental freedoms.

**Summary:**

The over-arching aim of relief of oppression is that, within the range of benefits negotiated over time with the local communities and organizations, an increasing proportion reflects a shared interest in improving participants' fundamental freedoms. We describe how harm reduction serves as a useful analogy for how we envision relief of oppression functioning in international research.

## Background

### How far do observational researchers' obligations extend?

On January 7, 2006, a prominent journalist, Stephanie Nolen, published a story in the *Globe and Mail*, a national Canadian newspaper claiming that Canadian scientists and other Western researchers studying the immunological response to HIV in a 25 year cohort of sex workers in Nairobi, Kenya may have exploited the women of the cohort by failing to remove them from the sex trade over the course of their participation in the research [1]. Nolen's article implied that part of the researchers' obligation to the women was to provide them with a viable alternative to the sex trade. She noted that the researchers had dramatically reduced the prevalence of sexually transmitted infections in the community, facilitated free basic health care and counseling for the participating women, and more recently access to anti-retroviral drugs for those infected with HIV, but also discussed how the women's continued participation in the sex trade, and thus in the cohort study, was of obvious benefit to the careers of the researchers. Nolen contrasted the tens of millions of dollars in research funding won over the years by the investigators for research on the cohort with the U.S. $0.80 per sexual encounter that the sex workers, like Ms. Simon, a 44 year old grandmother and cohort member for more than 20 years, rely on to eke out a meager existence in Nairobi's Majengo slum, where the research clinic is located [1].

Nolen's criticism of the Majengo STD Cohort study arose during a period of intensive debate about the obligations of researchers from high-income countries (HIC) conducting research in low and middle-income countries (LMIC), about what constitutes exploitation under these circumstances, and what can be done to avoid it. Commentators in this debate referred frequently to the U.S. Public Health Service Syphilis Trial--commonly known as the Tuskegee trial--as a paradigm example of exploitation, in part because the trial compounded the oppression of the poor black men who participated in the trial by allowing their medical condition to deteriorate even after effective treatment became available. This exacerbated their poverty and social deprivation [2]. A central question in that debate is whether investigators and sponsors from HIC have obligations to address background conditions of injustice in the communities in which they conduct their research, beyond the healthcare and other research-related needs of participants, to aspects of their basic life circumstances, such as obligations to provide alternative employment for Ms. Simon and her cohort colleagues. The Nairobi STD cohort case also extends the debate about justice in international research ethics beyond clinical trials into long-term observational research.

In this paper, we describe the Majengo STD Cohort study and the problem it poses for current thinking about exploitation and justice in international research. We then sketch the basic features of a new approach to understanding and operationalizing obligations of observational researchers, which we call "relief of oppression".

## Discussion

### The Majengo STD Cohort Study

The Majengo sexually transmitted disease (STD) Cohort study is a prospective, observational cohort study of STDs in sex workers in Nairobi, Kenya. The program was established more than 25 years ago as an exchange program between the departments of medical microbiology at the University of Nairobi and the University of Manitoba. In 1981, one of us (FP) was invited to begin conducting research in the Special Treatment Clinic of the Nairobi City Council in inner-city Nairobi [3].

In 1984 the STD Program was formally recognized by the World Health Organization (WHO) and designated as a WHO Collaborating Centre for STDs. Shortly before the WHO designation, Dr. Elizabeth Ngugi, a senior nursing sister in the Kenyan Ministry of Health, initiated a "community-based self-improvement program" among sex workers in the Pumwani area of Nairobi, which also facilitated research into the control of STDs in this community [3]. In 1985, the Program identified the spread of HIV among the sex worker cohort and designed what are now known to be foundational studies to understand the epidemiology of HIV and risk factors associated with its spread, including the identification of a cohort of sex workers who remained uninfected by HIV despite long-term exposure to the virus through sex with infected clients [3].

Paradoxically, there are potential harms to women who make unsuccessful attempts to leave the sex trade. One of the key scientific findings arising from the Majengo cohort has been that women who take breaks from sex work--for example, to visit family or to pursue alternative employment opportunities--temporarily stop their exposure to HIV and rapidly lose their immune status, which significantly increases their risk of HIV-infection if they resume sex work [4]. And so, short-term or incomplete efforts to secure alternative sources of income might, in the long-run, prove to be disastrous for these women.

Prior to the now common community advisory boards in research in LMIC, the Majengo STD Program established linkages with the community through elected peer leaders, who engaged in dialogue and negotiations with investigators about terms and conditions for participation in the research. Over time, these activities helped to develop and formalize a community among the women in the cohort that had not existed prior to the research and which has strengthened the women's collective voice on issues such as demanding condom use among clients, reducing discrimination against sex workers in the healthcare system and other public services, and reducing harassment of sex workers by the police [3]. These activities are consistent with much more recent initiatives and interventions among sex workers and their clients [5], such as community-led initiatives among women in the Sanagachi cohort studies in India, in which the women have taken collective action to tackle problems related to access to clean water and rescuing girls who have been sold into slavery [6].

The scientific success of the Majengo cohort, and the feasibility of some of the enhanced clinical services, particularly those for the treatment of sexually transmitted infections, also depended on local capacity to conduct the necessary clinical examinations and laboratory tests. The investigators worked closely with individual Kenyan clinicians, scientists and social workers, as well as with key institutional partners, most notably the University of Nairobi and the Kenyan Medical Research Centre, to ensure that the necessary capacity could be established and sustained in Nairobi. The durability of the cohort was also facilitated by extensive clinical and research exchange programs between trainees at the University of Manitoba and the University of Nairobi [3], and more recently by an infrastructure grant from the Canadian Foundation for Innovation [7]. These partnerships have also enabled a wide range of direct benefits for the women in the cohort and their communities, such as health education and the free distribution of condoms, which have likely averted thousands of HIV infections [8], and the provision of free treatment for a range of sexually transmitted diseases, as well as effective referral for care, such as hysterectomy, that might never otherwise have been available to the women.

Despite the range of positive contributions of the Majengo STD cohort study and the specific benefits to the women described above, Nolen's allegation of exploitation for failure to remove the women from the sex trade [1], and other recent critiques of the cohort [9], have prompted us to reflect further on these issues, particularly in light of the absence of explicit guidance for observational researchers.

### Avoiding exploitation in the Majengo STD Cohort study: what guidance?

'Exploitation' has proven to be an elusive concept in the context of international collaborative research [10]. According to Wertheimer, exploitation is localized in transactions and results from an unfair distribution of benefits between the exploiter and the exploitee [11]. The unfairness of the distribution of benefits in any given transaction must be determined, in advance of the realization of the actual benefits, according to a normative standard, which Wertheimer admits is "notoriously difficult to specify" [[11], p. 204].

This reasoning provides the underpinning for the "fair benefits" model, which holds that exploitation can be avoided by ensuring a fair distribution of the benefits agreed upon by sponsors, investigators and the population at risk of exploitation [12,13]. It recognizes that individuals and communities who participate in research have their own views about what constitutes appropriate and sufficient benefits in return for their participation, and should have a say in determining what will count as a fair and reasonable benefits in negotiations about a proposed study [12,13].

Wertheimer argues that: "...it is absolutely crucial to distinguish between moral defects in [the exploitee's] background situation and moral defects in the transactions that occur within that situation. Justice relates to background situations, whereas exploitation relates to transactions." [[11], p. 205] Along these lines, Emanuel argues that "...the purpose of specifying the extent of the obligation to provide benefits to LMIC that participate in biomedical research projects is to minimize the possibility of exploitation by developed country researchers and sponsors. Such benefits are not meant to address underyling background global injustice." [[14], p. 727]

In contrast, London argues that this transactional approach to fairness serves to "screen out" precisely the kind of information that makes concerns about justice in LMIC relevant, namely the extent to which populations and host country communities may have arrived at their circumstances of poverty and deprivation through unjust treatment by their own authorities, and/or international institutions and relationships that are unfair and oppressive [15]. The result for London is that there are duties of rectification for these past wrongs and that researchers share these as a matter of their citizenship in Western democracies, which have had a strong hand in the international relations that have contributed to the "background conditions", an argument that has also been made previously by Benatar [16].

These accounts of what is required to avoid exploitation offer observational researchers, such as the Majengo cohort investigators, little practical guidance. The transactional focus of Fair Benefits suggests a preference for narrow, tractable benefits over broad and complex one, yet the most pressing needs of the Majengo cohort women--as an example--are profound, intimately related to the research, and inextricably rooted in the complexity of social injustice. London's approach, on the other hand, despite its explicit focus on social justice, is under-developed in terms of practical guidance for investigators. What is missing is a framework that straddles these two polar views, one that helps observational researchers reconcile their day-to-day experience with needs arising from injustice with the requirement to ensure that participants benefit from their contributions to research in fair and meaningful ways.

Below, we proposed a way to address this problem, which we call 'relief of oppression'. We emphasize that some of the pressing injustices routinely experienced by observational researchers give rise to humanitarian obligations of 'rescue', or assistance, which vary in strength with the extent of the relationships with their participants, the special capabilities of the researchers and the circumstances of their opportunity to provide assistance. Combined with the now widely recognized obligation to ensure research participants receive some benefits in return for their participation in research, relief of oppression offers a pragmatic strategy for explicitly addressing participants' needs that arise from injustice.

### Relief of oppression

'Relief of oppression' is an organizing principle, analogous to the principle of harm reduction (as we describe in greater detail, below) that is now widely applied in public health practice. Relief of oppression aims to help observational researchers working in conditions of injustice and deprivation to clarify their ethical obligations to participants. It aims to bridge the gap between a narrow, transaction-oriented account of avoiding exploitation and a broad account emphasizing obligations of reparation for historic injustices. Specifically, it focuses explicitly on efforts to ameliorate some of the effects of the background conditions that limit fundamental freedoms of research participants. This explicit focus is necessary to ensure that benefits for research participants, negotiated with investigators and sponsors, do not simply avoid needs arising from systemic injustice, which may be difficult to address, in favour of needs that are more easily met by investigators.

Relief of oppression embodies the substantive aim of a widely held moral intuition that poverty and global injustice are matters of great moral urgency. Many theorists have developed this intuition and expressed it in terms of obligations on the part of individuals and institutions in LMIC to ameliorate these conditions [15,17,18]. The practical force of these obligations has been limited by considerable disagreement about the precise requirements of these obligations for various actors in specific real-world circumstances. In the absence of perfect agreement on the nature and scope of the relevant obligations, we view 'relief of oppression' not as a philosophic theory to solve the problems associated with previous accounts of these obligations, but rather as a framework to help clarify and operationalize existing obligations to research participants.

We propose that the main value of relief of oppression might lie in focusing researchers' consideration of benefits on those that have some relevance to background conditions of injustice. And, in doing so, also elevate the priority of these benefits, in relation to any other types of benefit that might be considered and negotiated with participants, according to the degree to which the participating communities are constrained in their realization of fundamental freedoms. The over-arching aim of relief of oppression is that, within the range of benefits negotiated over time with the local communities and organizations, an increasing proportion reflect a shared interest in improving participants' fundamental freedoms.

Jennifer Hawkins has described how the widespread consternation about the ethics of placebo controlled trials in LMIC can be understood in terms of condemnation of investigators for "flouting" Good Samaritan obligations [19]. Importantly, Hawkins emphasizes that such obligations are *not *obligations of benefit, as is the case within the fair benefits framework, but rather obligations of rescue. According to Hawkins, the failure to provide "easy rescues" "communicates a deep disrespect for the humanity of those one fails to rescue." [[19], p. 495] While Hawkins' analysis arose in the context of clinical trials of life-saving drugs, it is also relevant, and perhaps more so, to the long-term relationships between researchers and participants in observational research. More particularly, it is relevant to background conditions of injustice because it is precisely those conditions that so often give rise to the health and social circumstances that interest observational researchers working in global public health [20]. The concept of "rescue" is broad enough to permit a wide range of applications, from dramatically emergent circumstances, such as plucking a drowning person from the sea, to similarly dramatic but slightly less time-dependent challenges, such as clearing the area surrounding a local clinic of landmines, to arranging access to life-saving drugs, to assisting with the construction of shelter for communities displaced by conflict.

The "rescues", or humanitarian assistance, we have in mind are those associated with constraints on freedoms--our working conception of oppression. More specifically, we draw on Amartya Sen's "development as freedom" argument, in which he views 5 fundamental freedoms in society--political freedoms, economic facilities, social opportunities, transparency guarantees, and protective security--as being constitutive of development [21]. On Sen's account, development is essentially an accumulation of fundamental freedoms (or removal of oppression, or obstacles to human freedom). And so, the obligation of relief of oppression we advocate here is, in essence, to focus on those benefits that are most likely to contribute to this goal. For example, where a straight fair benefits analysis might view the construction of a new clinic in a village as sufficient benefit for the community in return for their participation in a study of HIV epidemiology, relief of oppression might require in addition the assurance that the clinic will not deny services to, or otherwise discriminate against, sex workers, or other marginalized groups within the community involved in the research and in need of care, a situation that arises frequently in low-resource settings.

Our view is consistent with the social justice foundations for public health and public health policy articulated recently by Powers and Faden [18], which is particularly relevant for observational research in global health. The social justice view rejects the "separate spheres" view of justice and argues that justice in public health cannot be set aside from "...how other public policies and social environments are structured or...how people are faring with regard to the rest of their lives." [[18], p. 10] As well, since the commensurability of benefits (e.g., provision of treatment for sexually transmitted infections for members of the study cohort vs. the range of benefits that emerge for a researcher who receives a multi-million dollar grant) is a major challenge for the implementation of the Fair Benefits approach, relief of oppression offers a mechanism to forge agreement around fundamental and shared interests [22].

Hawkins recognizes that the problem of how to assign moral responsibility for engaging in humanitarian assistance becomes complicated when there is more than one person in a position to assist, and when the goals of assistance are increasingly complex, as one would have to characterize removing women from the sex trade under the circumstances of the Majengo STD Cohort study. Unlike clinical research situations in which investigators' clinical training might make them uniquely qualified to make a medical intervention (e.g., if a participant suffers a heart attack during a clinical trial), in the case of a complex social challenge, like the removal of women from the sex trade, it is not clear that investigators have the expertise and experience to take on such a complex intervention, or even to fairly assess the full range of its implications. Hawkins recognizes the difference between what she calls an "easy rescue" and one that requires greater effort, risk and specialized skills, the latter obligation being weaker, accordingly [19].

### Operationalizing 'relief of oppression': the analogy of harm reduction

A recent Lancet review of harm reduction strategies for sex workers identifies 7 domains of potential harm routines experienced by sex workers: drug use, disease, violence, discrimination, debt, criminalization, and exploitation (including child prostitution, trafficking for sex work, and exploitation of migrants). The review also identifies a wide range of harm reduction strategies and interventions identified through decades of civil society initiatives, public health practice and research [23]. These include: education, empowerment, prevention, care, occupational health and safety, decriminalization of sex workers, and human rights-based approaches, peer education, and training in condom-negotiating skills [23]. The result is an empirically grounded framework of harm reduction strategies and the accumulated evidence of their effectiveness.

Harm reduction serves as a useful analogy for how we envision relief of oppression functioning in international research. As part of their normal deliberations and negotiations about benefits associated with participating in a prospective cohort, or other observational study, investigators and communities would identify specific obstacles and barriers in Sen's five domains of human freedoms (political freedoms, economic facilities, social opportunities, transparency guarantees, and protective security) and would agree on strategies aimed at removing or reducing these barriers. As with all other proposals currently being considered in this area, relief of oppression would require collective judgement about what proposed distributions of benefits between research participants and investigators should be considered fair. But, by requiring that at least some of the benefits--ideally in proportion to the perceived oppression of the participants and their communities participating in the research--be focused explicitly on relieving oppression, which should under most circumstances be a shared interest of both the host communities and the researchers, relief of oppression may also help to avoid the problem of how to reconcile the incommensurability of benefits to investigators compared to participants and communities in determining the fairness of a proposed distribution.

Another aspect of relief of oppression that should be considered in application is the length and depth of the relationship between researchers and research participants. Longer and deeper relationships increase the investigators' understanding of the needs of research participants arising from oppressive background conditions of injustice, increase the opportunities to provide assistance, and generally strengthen the human bonds between the investigators and the research participants. Accordingly, investigators' obligations to relieve oppressive background conditions increase over time. For example, a short-term observational study might focus exclusively on health service benefits, while a decade-long cohort study might also focus on economic benefits for the participants in terms of skills training and other employability measures. The precise scope of obligations arising from relationships is a matter of considerable debate. We agree with Dickert and Wendler that "hard and fast" rules are unlikely, but that "shared expectations and norms...may well evolve over time"[24], a process that we believe would be greatly facilitated by our analogy to harm reduction.

In this vein, we think that further clarification of the operations of relief of oppression will need to come from experience with its application, in precisely the same way that the concept of 'harm reduction' has been brought to life through years of practical experience. For example, rather than advocating detailed policies or strategies of application, a 1995 editorial in the American Journal of Public Health argued that "failure to use research findings" to evaluate the impact of harm reduction strategies to reduce the negative impact of illicit drug use "would violate the core value of a realistic pragmatism", and that "(t)he value of harm reduction policies should be assessed against their actual effects on drug-related harms rather than on their consistency with cultural traditions."[25] In the case of relief of oppression, we expect that it would be virtually impossible, in advance, to anticipate how the principle might be applied in any specific context, for example in routine observational studies to track the evolving epidemiology of HIV in sub-Saharan Africa and Asia, but we believe that the discipline of scientific reporting on these experiences could move this important dimension of research ethics beyond philosophic debate alone. For example, case studies in various observational research contexts would help to reveal creative interpretations of 'relief of oppression' and identify good practices and concrete strategies, tailored to specific contexts. Collectively, over time, the resulting insights would reveal trends in practice and outcomes and thereby provide researchers and communities with potential pathways for action.

As well, although we have used the Majengo cohort to illustrate the problem that relief of oppression aims to address, we view relief of oppression as broadly applicable in global health research, for example in research on childhood malnutrition or environmental health. These, and many other, research contexts reflect a disproportionate disease burden in poor populations, where various forms of oppression are likely to be at work, and both are often studied using long-term prospective cohort designs.

### Potential objections to relief of oppression

We anticipate two main objections to our proposal. First, we expect that some commentators will claim that relief of oppression turns observational researchers into humanitarian aid workers. This concern has been a central tension in the on-going debate about the scope of researchers' obligations to research participants, especially for research conducted in low and middle-income countries [10,24,26]. Relief of oppression will likely be viewed as too demanding on researchers by those who have argued for narrow obligations and as too forgiving by those who have argued for a more activist approach with respect to injustice. Our aim is to claim some currently barren territory in the middle ground of this debate by facilitating more focus on addressing injustice explicitly through the familiar and now widely accepted mechanism of benefit sharing. As well, we believe that Hawkins' account of humanitarian assistance provides a useful rubric for calibrating the level of assistance required according to urgency, opportunity, capability and the complexity of the aims of any proposed form of assistance or benefit.

The second likely objection is that relief of oppression is too vague a concept to provide concrete guidance. If our aim was to develop a robust philosophic account of observational researchers' obligations, this objection might carry some weight. But our aim is fundamentally a pragmatic one, namely to emphasize--in line with Hawkins--that the relevant obligations are humanitarian, and not primarily obligations of benefit, but at the same time recognize that providing humanitarian assistance that is focused on the promotion of fundamental freedoms, and therefore the relief of oppression, is compatible with normal benefit sharing processes and practices. As well, for our proposal to have impact, it is also important to recognize--as has been the case for harm reduction--that there is an important distinction between a concept that is still poorly specified due to lack of experience with creative application, from one that is genuinely too vague to provide meaningful direction. Like harm reduction, the core idea of relief of oppression is readily understandable, but the full potential of its impact will be known only through creative application and evaluation.

## Summary

The case of the Majengo STD Cohort illustrates that current accounts of how to avoid exploitation in clinical trials in LMIC provide inadequate guidance for observational research, where there is often no opportunity for direct benefit from a specific intervention, and where the depth and complexity of needs arising from injustice are often intimately related to the research. We advocate an approach that recognizes obligations of "rescue" or humanitarian assistance focused on improving fundamental freedoms for participants, which we have called relief of oppression. This approach facilitates explicit attention to background conditions of injustice in society and encourages observational researchers to include the benefits of this type of assistance among the range of benefits negotiated with participating communities, rather than avoiding them in favour of benefits that are easier to achieve. We believe that harm reduction provides an ideal analogy for how relief of oppression can be implemented, and refined over time, to ensure that the types of benefit it encourages result in demonstrable relief of oppression for participants of observational research in LMIC.

## Competing interests

The authors declare that they have no competing interests.

## Authors' contributions

JVL conceived the paper and led the drafting of the manuscript; SB, JK, REGU, FRP and PAS reviewed and provided critical input on each draft of the manuscript.

## Pre-publication history

The pre-publication history for this paper can be accessed here:

http://www.biomedcentral.com/1471-2458/10/384/prepub

## References

[B1] Nolen S Sex slaves for science The Globe & Mail. Toronto 2006 Jan 7. F4

[B2] Angell M The ethics of clinical research in the Third World New England Journal of Medicine 1997 337 847 849 10.1056/NEJM199709183371209 9295243

[B3] Ronald A others The Nairobi STD Program: An international partnership Infectious Disease Clinics of North America 1991 5 2 337 352 1869813

[B4] Kaul R others Late seroconversion in HIV-resistant Nairobi prostitutes despite pre-existing HIV-specific CD8^+ ^responses Journal of Clinical Investigation 2001 107 3 341 349 10.1172/JCI10714 11160158 PMC199193

[B5] Ngugi Elizabeth N Branigan Erin Jackson Denis J Gibney Laura, DiClemente Ralph, Vermund Sten Interventions for commercial sex workers and their clients Preventing HIV in Developing Countries: Biomedical and Behavioral Approaches 1999 New York: Plenum Press 205 230

[B6] Joint United Nations Programme on HIV/AIDS Female sex worker HIV prevention projects: Lessons learnt from Papua New Guinea, India and Bangladesh 2000 Geneva: UNAIDS

[B7] Canadian Foundation for Innovation grant Canada-Kenya International Collaboration on Infectious Diseases Research: Building on Strengths and Enhancing Capacity for Innovation 2002 Francis Plummer, University of Manitoba http://www.innovation.ca/docs/projects/CFIawards230410.xls

[B8] Ngugi EN others Prevention of transmission of human immuno-deficiency virus in Africa: Effectiveness of condom promotion and health education among prostitutes Lancet 1988 2 887 890 10.1016/S0140-6736(88)92480-4 2902326

[B9] Andanda P Vulnerability: Sex workers in Nairobi's Majengo slums Cambridge quarterly of health care ethics 2009 18 2 138 146 10.1017/S0963180109090239 19250567

[B10] Exploitation and Developing Countries Hawkins Jennifer, Emanuel Ezekiel J The Ethics of Clinical Research 2008 Princeton: Princeton University Press

[B11] Wertheimer Alan others Emanuel Ezekiel J Exploitation in clinical research The Oxford Textbook of Research Ethics 2008 New York: Oxford University Press 201 210

[B12] The participants in the 2001 Conference on Ethical Aspects of Research in Developing Countries Fair Benefits for Research in Developing Countries Science 2002 298 2133 4 10.1126/science.1076899 12481120

[B13] The participants in the 2001 Conference on Ethical Aspects of Research in Developing Countries Moral standards for research in developing countries: From "reasonable availability" to "fair benefits" Hastings Center Report 2004 34 3 17 27 10.2307/3528416 15281723

[B14] Emanuel Ezekiel J Emanuel Ezekiel J Benefits to host countries The Oxford Textbook of Research Ethics 2008 New York: Oxford University Press 719 728

[B15] London AJ Justice and the human development approach to international research Hastings Center Report 2005 35 1 24 37 10.1353/hcr.2005.0009 15799498

[B16] Benatar SR Reflections and recommendations on research ethics in developing countries Social Science & Medicine 2002 54 1131 1141 10.1016/S0277-9536(01)00327-6 11999507

[B17] Nagel T The problem of global justice Philosophy & Public Affairs 2005 33 113 147 10.1111/j.1088-4963.2005.00027.x

[B18] Social Justice: The Moral Foundations of Public Health and Health Policy Powers Madison, Faden Ruth 2006 New York: Oxord University Press

[B19] Hawkins JS Justice and placebo controls Social Theory and Practice 2006 32 3 467 496 17225338 10.5840/soctheorpract200632322

[B20] WHO WHO Commission on Social Determinants of Health Report 2008 http://www.who.int/social_determinants/final_report/en/1-1-2010

[B21] Sen Amartya Development as Freedom 1999 New York: Anchor Books

[B22] London AJ Two dogmas of research ethics and the intergrative approach to human subjects research Journal of Medicine and Philosophy 2007 32 99 116 10.1080/03605310701255727 17454417

[B23] Rekart ML Sex-work harm reduction The Lancet 2005 366 9503 2123 35 10.1016/S0140-6736(05)67732-X 16360791

[B24] Dickert N Wendler D Ancillary care obligations of medical researchers JAMA 2009 302 4 424 428 10.1001/jama.2009.1076 19622821

[B25] Des Jarlais DC Editorial: Harm reduction--a framework for incorporating science into drug policy American Journal of Public Health 1995 85 1 10 12 10.2105/AJPH.85.1.10 7832242 PMC1615258

[B26] Lavery JV Putting international research ethics guidelines to work for the benefit of developing countries Yale Journal of Health Policy, Law, and Ethics 2004 4 319 36 15536914

